# Supplementary material for: Are carbon emissions trading and green financial instruments synergistic? -Comprehensive quantitative research based on content analysis
Source: PLoS One. 2024 Mar 7;19(3):e0298601. doi: 10.1371/journal.pone.0298601 (PMC10919601; doi:10.1371/journal.pone.0298601)
Supplement: S1 Data — (ZIP) [file pone.0298601.s001.zip › Data/地级市Z_F工作报告.zip!/║■▒▒/╩«╤▀╩╨/└┤╫╘╣1⁄2╓┌║┼í╛┬φ┐╦╩2╛▌═°í┐_2007.docx]

各位代表：

　　现在，我代表市人民政府向大会作工作报告，请予审议，并请各位政协委员和列席会议的同志提出意见。

　　一、２００６年工作回顾

　　２００６年，全市实现生产总值３４５亿元，同比增长９％；地方一般预算收入１３．２亿元，增长１６％；全社会固定资产投资１２２．１亿元，增长１９．９％；利用外资３４５９万美元，增长８６．１％；外贸出口３９２１万美元，下降４９．１％；社会消费品零售总额１４４．７亿元，增长１５．７％；居民消费价格涨幅低于４％；城镇居民人均可支配收入７７１８元，增长７．２％；农民人均纯收入２１９１元，增长１０．１％；万元生产总值综合能耗降低４％，城镇登记失业率、人口自然增长率控制在省定目标以内。除生产总值增幅低于计划一个百分点、外贸出口比计划少２１００万美元外，其他指标均完成或超额完成了计划。

　　（一）新农村建设稳步推进，县域经济快速发展。围绕新农村建设，加大“三农”工作力度，努力促进农业增效、农民增收、农村发展，第一产业实现增加值３９亿元，增长８．２％。编制了全市新农村建设总体规划，启动了试点示范村建设。粮食生产创８年来最好水平，粮食总产９６．１万吨，增长２１．５％；油料总产７．５万吨，增长７．５％。特色产业不断壮大，新增基地２０万亩，特色农产品出口创汇１０００万美元。劳务经济成为农民增收的重要渠道，加强农村劳动力培训工作，完成各类培训５３．２万人次；外出务工农民达５７．３万人，增长１４．１％；务工收入２７．１亿元，增长１８．１％。农民生产生活条件明显改善，建设通村水泥路２８８０公里，新建沼气池１．６６万口，解决６．２５万人饮水困难。扶贫开发深入推进，不得温饱人口减少４万人，低收入人口减少５万人，完成扶贫搬迁２７８５户１．１４万人、地质灾害搬迁７１４户２７６４人，改造农村危房３２４７户。县域经济发展明显提速，五县一市实现生产总值１３５．３亿元，增长１１．４％，高出全市２．４个百分点。

　　（二）工业经济稳步回升，新的增长点活力显现。进一步支持东风公司优化产品结构，适时调整工业发展思路，以工业园区为载体，重点发展整车和零部件产业；以优势资源为依托，大力发展特色工业；以重大项目为支撑，提升产业规模和发展水平。地方工业自主发展能力进一步提高，市县属工业增加值增长２６．６％，占全市工业的比重达３９．５％，提高了８．７个百分点，特别是地方汽车工业增速达３７％，医药化工、有色金属、橡胶制品、纺织、饮料等行业均增长 １５％以上，信息产业增长４７％，民营工业增长３７．３％；十堰经济开发区工业增长１３３．８％，东城、西城开发区分别增长４２．８％、２７．３％。地方工业的强势增长，带动了全市工业稳步回升，全市规模以上工业实现增加值１２０．８亿元，比年初回升了４５．４个百分点。工业经济运行质量和效益明显提高，全市规模以上企业新增６２家；规模以上企业实现利润１８．２亿元，增长５％；１２项产品获省名牌产品称号，车神牌离合器、长彬牌减振器系列产品首次成为国家免检产品。一批新的增长点正在成长壮大，东风中客项目正式落户十堰，东风渝安微型车加速扩张，神河万辆低速汽车项目基本建成，三环专汽３万辆整车项目即将实施，双星东风轮胎全面恢复生产；万向通达工业园７万平方米标准厂房全部建成，普林工业园二期、温州工业园建设顺利推进，上海圣奥化工等项目入驻西城开发区。

　　（三）旅游产业增速较快，消费市场日趋活跃。武当山游客服务中心投入使用，景区实行封闭管理；第七届武当国际旅游节成功举办，以武当山为龙头的旅游经济发展势头强劲。全市接待游客、旅游总收入、旅游创汇分别增长１１．５％、１１．２％和７１％，武当山门票收入增长５０．４％。城乡市场共同繁荣，城市实现零售总额增长１７．２％，县及县以下增长１３．４％。新增连锁超市３６家、乡镇超市（店）２６家、村级超市（店）３１２家。

　　（四）项目建设顺利推进，基础设施进一步完善。水电建设取得重大突破，全市新增发电能力１５万千瓦，鄂坪电站、松树岭电站并网发电，竹山２２０千伏输变电工程建成。丹江大坝加高工程全面开工，南水北调丹江口施工大桥建成通车。交通建设步伐加快，襄渝铁路二线、十漫高速公路建设顺利推进；改造干线公路１７７公里，新建省际出口路及经济断头路１５４公里；房县城关至温泉段一级路基本竣工，六两路改造完工，将军河大桥建成通车。城镇建设成效显著，天津路、重庆路建成通车，白浪路、武当路改造工程基本完工，浙江路、甘肃路、许白路工程建设进展顺利，主干道循环路网日趋完善，新增城市发展空间２０平方公里左右；游泳馆、美术馆建成开馆；张湾市场、老虎沟市场投入使用；新建公厕１１个、停车泊位２０９５个；实现了重庆路、天津路、武当路保洁权的市场化运作，对城区渣土处置管理逐步规范；全市有７个镇、７５个村列入省“百镇千村”示范工程。

　　（五）各项改革全面深化，对外开放取得新进展。争取企业改制政策性贷款和贴息贷款２亿元；８家市直重点企业和华阳集团完成了改制任务；东风轮胎集团实现了政策性破产，化解金融债务１１．４亿元。乡镇综合配套改革进一步深化，初步建立了“以钱养事”机制。财政与编制政务公开全面推进，５２０个部门预算单位、所有编制单位实现了网上公开。参加了广交会、青洽会、鄂港经贸洽谈会等招商引资活动，加强了签约项目的跟踪服务，全市实际利用外资超额完成省定任务，利用市外境内资金增长１１％。

　　（六）财政收入快速增长金融运行平稳健康。全市地方一般预算收入１３．２亿元，增长１６％；财政一般预算支出４１．５亿元，增长２４．４％。全市金融机构各项存款余额５１０．５亿元，比年初增加６６．４亿元；各项贷款余额２４７．１亿元，比年初增加５０．９亿元。贷款投放增长较快，贷款增量占存款增量的７６．７％。我市被省政府授予Ａ级金融信用市。

　　（七）社会事业全面发展，社会大局保持稳定。全市科技三费增长１２．５％，申请专利增长３２％，省级高新技术企业新增８家，高新技术增加值增长１０．５％，我市被列入国家第二批“科技强警示范城市”建设。全市小学和初中适龄儿童入学率分别达到１００％和９６．６％，普通高考上省线人数增长１９．４％；落实农村义务教育阶段“两免一补”对象１４．６万人，比上年增加５．７万人；改扩建农村学校２２所；茅箭区成为省政府首批命名的“人民满意教育县（市、区）”。新型农村合作医疗覆盖７个县市区；重点建设和改造了 １２个乡镇卫生院、５５个村级卫生室。武当武术、武当宫观道乐、伍家沟民间故事列入第一批国家级非物质文化遗产；加强网吧市场整治，率先在全国实现“零点断网”。成功举办了市第三届全民健身运动会。广播电视人口有效混合覆盖率达到９５％。计划生育实现省定目标。《丹江口水库及上游水污染防治和水土保持规划》得到国家批复，环保能力建设进一步加强。全市城镇新增就业３．６９万人，下岗失业人员再就业１．８万人，社会保险新增扩面６．７７万人。城镇低保对象实现了应保尽保，五保对象基本实现了集中供养，１７００名农村分散供养的孤儿全部纳入了救助范围，享受农村特困救助人口新增２．４万人，城乡大病医疗救助全面启动。住房公积金归集率达８０％以上。深入推进“平安十堰”建设，组织开展了“严打”整治活动，建立了公共突发事件应急体系。大力实施“安全生产年”活动，安全生产形势总体平稳。认真处理信访突出问题和群体性事件，信访总量下降３１％，集体访下降３．３％，进京非正常上访下降３０％。

[FS:PAGE]

　　（八）民主法制建设扎实推进，精神文明建设取得新成效。市政府坚持定期向人大报告工作、向政协通报情况，重大决策征求民主党派、党外人士、人民团体意见，办理人大代表议案、建议１６２件，办理政协委员提案３５１件，办复率１００％。坚持做好民主评议政风行风工作，对五家公用企事业单位进行了集中评议。坚持依法行政，启动了“五五”普法规划，组织实施《公务员法》，基本完成了公务员登记工作。市长热线受到群众的广泛信任，全年共接听群众来电３万多件，反馈率１００％。认真开展治理商业贿赂工作，立案４３件，处理５１人。国防动员、人民防空、民兵预备役和优抚安置工作取得新成绩，争创“全国双拥模范城”五连冠工作进展顺利，市审计局被中央文明委授予“全国精神文明建设工作先进单位”，武当山被授予省级文明风景旅游区称号。

　　这里着重报告去年“十件实事”进展情况。在市三届人大二次会议上，市政府向全市人民郑重承诺，着力办好十件实事。一年来，市政府克服种种困难，确保了十件实事顺利推进，解决了一批市民关注的热点难点问题。计划当年完成的安置就业和社会保险扩面、新建公厕和停车泊位、减少贫困人口、扩大“两免一补”对象、农村劳动力各类培训、农村危房改造、对分散供养孤儿实行救助、农村医疗卫生条件改善、“万村千乡”工程、乡村油路、张湾市场和老虎沟市场建设等实事全部完成或超额完成任务；跨年度的鄂西北小商品市场建设、白浪至许家棚一级路建设，已按年度计划推进到位，今年将全部建成。

　　在全市三个文明建设中，外事侨务、民族宗教、档案、邮政、电信、地震、气象、水文、对台、老龄、残联、地方志、机关事务管理等方面的工作都取得了新的成绩。

　　各位代表，上述成绩的取得，是市委正确领导、全市人民团结拼搏的结果，是市人大、市政协监督支持的结果。在此，我代表市政府，向所有参与、支持、关心十堰发展的同志们、朋友们表示衷心的感谢和崇高的敬意

　　在看到成绩的同时，我们也清醒地认识到，前进道路上还有许多困难和问题。主要是：有竞争力的企业和产品较少，重大生产性项目不多；农业基础脆弱，农民持续增收难度加大，农村生产生活条件亟需改善；能源等消耗水平仍然过高，资源和环保压力进一步增大；财政减收增支因素增多，中小企业融资渠道不畅；政府自身改革和职能转变还不到位，公务员素质和政务效率还不高，还存在着作风不实和腐败等现象。对于这些困难和问题，我们将在今后工作中认真解决。

　　二、２００７年工作思路和主要任务

　　２００７年是实现“十一五”规划、推进和谐十堰建设的重要一年。新的一年，我市发展的政策环境更加优越，经过多方争取，我市成为同时享受西部大开发和振兴老工业基地政策的地区，被列为全国４５个公路枢纽城市之一，国家对新阶段扶贫开发和新农村建设的投资力度将进一步加大，《丹江口库区经济社会发展规划》国家正在编制之中、年内可望出台；重点工程对经济发展的拉动更加强劲，南水北调中线工程加快推进，十漫高速公路年内建成通车，襄渝铁路二线投资建设进入高峰期；东风公司立足于“中国第一、世界前三”的发展定位，进一步推动东风商用车事业发展，加大对零部件项目的投入和技术改造，东风公司对地方经济带动作用将进一步加强；“双亿工程”全面实施，县域经济持续快速发展，城乡消费市场日趋活跃，经济自主增长的潜能将进一步释放。我们要抢抓发展机遇，沉着应对挑战，扎实做好工作，满怀信心地迈入环境更优、基础更牢、效益更好、速度更快的发展新阶段。

　　根据当前形势，今年我市经济社会发展的总体要求是：以邓小平理论和″三个代表″重要思想为指导，全面落实科学发展观，认真贯彻市第三次党代会精神，围绕巩固汽车城地位、服务南水北调、推进新阶段扶贫开发三大历史任务，深入实施工业兴市、生态立市和人才强市战略，着力调整经济结构和转变增长方式，着力加强资源节约和环境保护，着力推进改革开放和自主创新，着力促进社会发展和解决民生问题，努力实现经济又好又快发展，加快构建和谐十堰。

　　今年全市经济社会发展的主要预期目标是：生产总值增长１０％，地方一般预算收入增长１１％，全社会固定资产投资增长１８％，利用外资增长１０％，外贸出口总额增长１０％，社会消费品零售总额增长１３％，居民消费价格总水平控制在３％以内，城镇居民人均可支配收入增长１０％，农民人均纯收入增长６％，城镇新增就业岗位３．５万个，登记失业率控制在４．５％以内，人口自然增长率控制在省定目标以内，万元生产总值能耗下降４％，主要污染物排放总量减少２％。

　　根据上述总体要求和奋斗目标，今年重点抓好八个方面的工作。

　　（一）扎实推进新农村建设。

　　积极发展现代农业。依靠科技进步，推进产业化、规模化经营，提高农业综合生产能力。进一步调整农村产业结构，壮大特色经济规模，新建特色产业基地２０万亩以上；加大龙头企业的扶持力度，突破性发展农副产品加工业，大力发展各类专业合作经济组织。突出发展草食畜牧业，推进养殖小区建设，加快畜禽品种改良，加强疫病防控。加强水产基地建设，优化水产品结构。加强蔬菜基地建设，做大蔬菜产业。建立完善农业质量标准体系，积极打造有机农业品牌。

　　大力发展务工经济。以提高农村劳动力综合素质为目的，以竞争性技能培训和适应性培训为重点，以市县培训基地为依托，大力开展实用技术培训、务工技能培训、扶贫培训、就业培训，增强农村劳动力的就业创业能力。推进城乡劳务市场一体化建设，切实做好维权服务和信息引导工作。力争全年完成各类培训５７万人次，新增务工劳力７万人以上。支持打工能人回乡创业，形成农村人才双向流动机制。

　　扎实做好扶贫开发工作。全面完成２００６年１０７个重点村建设，启动１０９个重点村整村推进工作。减少不得温饱人口４万人，减少低收入人口５万人。加大老区乡村帮扶力度，着力改善老区群众生产生活条件。深入推进城区农村扶贫开发，进一步解决行路难、饮水难、通讯难问题。将重点村整村推进与新农村试点示范村建设结合起来，扎实做好１５３个试点示范村建设工作。深入开展市校科技合作、百企联百村等共建新农村活动。

　　加强农村基础设施建设。新建基本农田３万亩，新修改造塘堰１７６３口，完成４９座病险水库加固任务，推进防洪工程建设，新建农村沼气池１．５万口、沼气示范村３０个，更新节柴灶４万座，抓好通村水泥路、饮水安全工程建设和农村危房改造。

　　发展农村公共事业。免除农村义务教育阶段学生学杂费，对贫困家庭学生免费提供课本，继续加强农村寄宿制学校建设，对贫困寄宿生给予生活补助。全面推行新型农村合作医疗，启动３个县级医院、１４个乡镇卫生院等国债项目，加快村级卫生室建设。推进２０个乡镇综合文化站和１００个村文体设施建设。

　　深化农村改革。完善土地承包经营权依法、自愿、有偿流转机制，促进农村规模经营和结构调整。巩固农村税费改革成果，继续深化以乡镇机构改革、农村义务教育和县乡财政体制改革为主要内容的综合改革。稳步推进集体林权制度改革，严格按照“公开、公平、公正”原则和规范的操作程序，将林地使用权和林木所有权真正确权到户，进一步加强林权管理。逐步化解乡村债务，完善涉农收费公示制，确保农民负担不反弹。

　　（二）全力促进工业提质增量。

　　着力实施“双亿”工程。重点支持一批新增投资过亿元的工业项目、培植一批新增销售收入过亿元的骨干企业，市财政今年安排１０００万元，专项用于项目单位贴息补助和奖励。支持东风中客、三环专汽３万辆整车项目尽快建成投产，扶持东风渝安和双星东风的技改扩能项目建设。同时，加快推进正和车身、世纪中远、益民铜材、圣奥橡胶助剂、金鼎扩能改造等重点项目建设，继续支持高科技含量、高附加值的“双高”企业发展。

[FS:PAGE]

　　 强势发展工业园区。全面落实《支持工业园区发展的意见》，充分发挥国家汽车关键零部件产业基地的品牌效应，促进生产要素向园区集聚、优势企业向园区集中。支持开发区推行“一区多园”，发展特色工业园区。加快万向、三环、普林二期、温州汽配、西城化工、东风商用车物流等工业园区建设步伐。

　　着力支持整车企业发展壮大。鼓励具有整车生产资质的企业自主创新，尽快做大做强。引导整车生产企业优势互补，共同开拓市场。推动整车企业与零部件企业合作，促进整车与零部件良性互动。大力发展汽车电子、汽车模具产业，促进其与整车产业协同发展。

　　大力发展县域工业。全面落实支持县域经济发展的各项政策，支持县市因地制宜发展特色工业，做强优势产业。有序开发水电产业，继续抓好在建水电项目建设，争取开工建设潘口等一批新的水电项目，进一步做好孤山、龙背湾等项目的前期工作，完成城区电网结构优化工程。着力培植一批特色产品加工龙头企业，带动医药化工、绿色食品、矿产开发、建筑建材等产业快速发展。

　　切实做好服务企业工作。认真落实《关于进一步支持东风公司十堰基地企业发展的意见》，全面实施《“十一五”政企共建合作纲要》，进一步优化东风公司发展环境；加强与东风公司各板块的协调联系，支持重大项目建设，争取更多项目落户十堰。继续支持汉江集团、三环专汽等中央、省属企业加快发展。坚定不移地推进市属企业改制重组，对尚未完成改制的企业，继续实行专班负责，因企施策，尽快完成改制任务；积极稳妥地处理改制遗留问题，确保职工得到妥善安置、国有资产不流失。引导企业建立现代企业制度，加强技改和研发，提高营销管理水平，争创精品名牌。做好煤、电、油、运等重要生产要素的组织协调工作，努力为企业排忧解难。严格规范对企业的检查、评比、排序活动，切实减轻企业负担。

　　（三）大力推进第三产业提档升级。

　　加快发展旅游业。完成水体旅游规划编制。加快武当山重点旅游项目建设，积极开发县市旅游资源，形成主要景区连接互通的旅游线路，提升以武当山为龙头的旅游品牌。加强区域旅游合作，打造精品旅游线路，广泛开拓客源市场，力争旅游总收入增长１０％以上。

　　加快流通网络体系建设。做好重庆路商贸流通业发展规划，加快建设云燕农副产品博览交易中心，力争鄂西北小商品批发市场尽早投入使用；支持中国（十堰）汽配城创建品牌，扩大市场容量。加强市场价格监管，保持价格总水平基本稳定。

　　努力扩大外贸出口和劳务输出。着力培植一批产品科技含量高、市场竞争力强的出口型企业，支持东风进出口、汉江集团、东风实业、双星东风轮胎、聚达食品等骨干企业开拓国际市场。加快特色产品出口基地建设，扩大出口规模。加强公路口岸建设，提高通关能力。推进外派劳务基地县建设，积极开拓国际劳务市场。

　　大力拓展新兴服务业。优先发展信息应用、文化创意等知识密集型服务业，积极支持银行、证券、保险等资金密集型服务业扩展业务，着力培育通信、社区服务、休闲娱乐、医疗保健等消费热点，规范发展中介服务组织。

　　（四）保持投资快速增长势头。

　　切实做好项目工作。认真研究国家产业发展政策和重点支持领域，找准资源、产业与政策支持的对接点，筛选一批高质量的项目，调整充实项目库。加大项目申报力度，积极争取国家财政预算内投资、国债和银行信贷支持，广泛吸纳民间投资，重点建设一批大项目和生产性项目，进一步优化投资结构。实行重大项目专班负责制，加强协调服务，严格项目管理，提高重大项目资金到位率和开工竣工率。

　　加大招商引资力度。突出汽车、旅游、高新技术、无公害农业、矿产资源开发等招商重点，组织参与重大招商活动，提高招商引资效率，实现利用外资和引进市外境内资金同比增长１０％以上。鼓励企业、园区和开发区发挥主体作用，广泛寻求国际国内战略合作，主动承接发达地区产业转移和资金、技术、人才辐射。加大对现有外资企业的服务力度，支持企业增资扩股。

　　全力服务国家重点工程建设。认真做好协调服务和后勤保障工作，切实优化施工环境，支持丹江大坝加高、十漫高速公路和襄渝铁路二线等国家重点工程建设。落实移民扶持政策，精心做好库区移民、生态移民安置的前期工作，启动水源区生态环保项目。

　　（五）加强交通和城镇建设。

　　加快交通建设步伐。推进襄渝铁路二线建设，争取十漫高速公路建成通车，做好襄天、郧房高速公路立项争取工作。改造国省道干线路网９３公里，建设省际出口路和经济断头路１５０公里。做好风神大道、郧县汉江二桥的前期工作，加快许白路、柳树垭隧道建设步伐，争取龙王垭、雷峰垭、五条岭、土地岭等隧道开工建设。整治汉江航道，提高航运能力。

　　加强城镇基础设施建设。争取浙江路、甘肃路、丹江路和许白路全面建成通车，力争市博物馆、接待中心和行政中心等工程如期完工。加大“百镇千村”示范工程建设力度，确保重点镇和示范村建设取得实效。

　　提升城镇管理水平。启动新一轮城市总体规划的修编，高标准编制十堰东部新城概念规划。加大城镇环境综合治理和执法力度，确保市容市貌整洁有序。继续加强噪音污染和粉尘渣土污染治理，依法规范建筑市场。引导经营业主入店入场经营，整治违章占道现象。开展市容环卫示范区创建活动，以点带面，逐步推开。加快市政公用事业市场化进程，提高运行效率。

　　（六）做好财税金融工作。

　　切实提高依法理财水平。加大对开发区、工业园区和汽车产业、特色产业的扶持力度，努力扩大财源。坚持依法组织收入，确保主体税种稳定增长；推进“金财工程”建设，确保税收及时、足额、均衡入库。进一步优化支出结构，保障法定支出和重点支出需要。深化财政改革，扩大财政与编制政务公开范围，健全公共财政体系。

　　充分发挥金融对经济的支持作用。加强诚信十堰建设，优化投融资环境。引导金融机构加大对优势产业、优质项目、优秀企业的信贷支持，实现互利双赢。扩大与开发银行的合作，共建融资平台，拓展融资领域。发挥中小企业信用担保中心作用，帮助中小企业解决融资难题。引导金融机构加大对新农村建设的信贷支持力度。

　　（七）努力构建和谐十堰。

　　大力发展社会事业。实施农村义务教育经费保障新机制，切实做好“两基”迎接国家检查验收工作；优化城区教育资源，着力缓解中心区教育资源不足的矛盾；加快湖北汽院科技学院、郧阳医学院药检学院建设，推进郧阳师专扩建、职业技术学院迁建和市职业技术（集团）学校新校区建设。建立社区、乡镇公共文化服务体系，开展丰富多彩的文体活动，推进淹没区文物保护抢救工作；全面推进自然村“村村通广播电视”工程，争取广播电视人口有效混合覆盖率提高到９７％。健全公共卫生体系，提高公共卫生突发事件应急能力；加强国家级和省级重点专科、重点实验室建设，巩固提高区域医疗中心地位；发展社区卫生服务，建立城乡医院对口支援、大医院和社区卫生机构双向转诊、高中级卫生技术人员定期到基层服务制度。加速建立人口计生工作新机制，稳定低生育水平，提高出生人口素质，治理出生人口性别比偏高问题。

　　切实抓好就业和社会保障工作。全面落实就业和再就业优惠政策，鼓励自主创业和自谋职业，积极开发公益性就业岗位。继续扩大社保覆盖面，严格社保基金的监督和管理，增强社保基金支付能力。建立完善覆盖城乡的社会救助体系，提高城乡低保、特困救助标准，推行农村社会养老保险试点，建立农村最低生活保障制度。加强社区公共设施建设，拓展社区服务领域。坚持公交优先原则，发展公用交通事业。狠抓住房公积金归集扩面工作，规范发展个人住房贷款。积极支持老龄工作、社会福利事业和残疾人事业，大力发展慈善事业。

[FS:PAGE]

　　保障群众合法权益和生命财产安全。加强劳动保障监察执法，规范企业用工行为。逐步建立进城务工人员工伤、医疗等保险制度，改善务工人员居住环境。加强法律服务工作，建立和完善弱势群体的法律援助体系。完善信访、投诉等群众利益表达机制，重点解决土地征用、房屋拆迁、环境保护、企业改制、涉法涉诉中损害群众权益的问题。加大在工程建设、道路交通、矿山开采等薄弱环节的安全生产执法力度，强化食品、药品、餐饮监管。加强社会治安综合治理，健全群防群治防控体系，扩大视频监控覆盖面。

　　加快建设资源节约型、环境友好型社会。以资源节约、综合利用、清洁生产为重点，加快发展循环经济。严格执行土地利用总体规划和年度计划，合理开发利用土地资源。继续推进退耕还林和天然林保护，确保治理水土流失面积１１０平方公里。切实执行环保法律法规，加强工业重点行业污染治理，确保黄姜生产企业如期达标排放、神定河污水处理厂正常运转。

　　深入开展精神文明创建活动。继续推进“文明十堰”建设，做好新一轮国家、省级文明城市创建工作。深化“十星级文明农户”创建活动，建设文明新村。加强国防教育和民兵预备役工作，认真落实军转干部、复退军人安置政策，支持部队和国防建设，深入开展“双拥”活动，巩固军政、军民团结。

　　（八）强力推进政府自身建设。

　　进一步转变政府职能。深化国有资产管理体制、投资管理体制改革，加快政府公共事务的社会化和市场化改革，推进政企分开、政事分开和政社分开。积极为各类市场主体提供良好服务，创造公平竞争环境。强化政府社会管理和公共服务职能，注重解决民生问题。

　　坚持科学民主决策。自觉接受人大监督、政协民主监督和社会监督、舆论监督，完善重大事项集体决策制度、专家咨询和评估制度、决策听证和公示制度、决策责任追究制度，提高决策水平。全面推行政务公开，方便群众办事和监督。

　　切实依法行政。扎实推进‘五五’普法工作，深入贯彻《全面推进依法行政实施纲要》，严格依照法定权限和程序行使权力、履行职责，提高政府执行力和公信力。理顺执法体制，坚决纠正多头执法、执法不公甚至执法违法行为。深入贯彻《行政许可法》，进一步减少和规范行政审批事项，简化和规范审批程序，切实提高审批效率。

　　加强政风建设。深入开展政风行风评议工作，增强机关优质服务意识，提高政务效能和服务水平。强化行政服务中心、招投标中心、采购中心和经济环境投诉中心等窗口单位功能，进一步加强管理，完善制度，规范和创新行政管理流程，加快电子政务建设，降低行政成本，提高行政效能。进一步完善目标管理责任制，加强督办检查，确保政令畅通，促进工作落实。认真落实廉政建设责任制，继续抓好公务员特别是领导干部的教育、监督和廉洁自律，继续抓好大案要案查处，继续抓好纠正损害群众利益的不正之风，继续抓好源头治理、推进体制机制创新，推动反腐倡廉工作深入开展。

　　严格责任追究制度。加快建立行政问责制，把行政问责与行政监察、审计监督结合起来，有责必问，有错必究。积极开展绩效评估，将评估结果作为行政问责的依据。按照奖优、治庸、罚劣的原则，充分发挥绩效评估的激励约束作用，坚决反对虚报浮夸、急功近利，反对搞劳民伤财的形象工程和政绩工程。

　　各位代表，今年市政府将继续办好十件实事，着力解决当前人民群众最关心、最直接、最现实的利益问题。（１）全市城镇新增就业３．５万人，社会保险扩面新增６万人。（２）建立廉租住房保障制度，对城区人均住房８平方米以下的特困户实行应保尽保。（３）农村特困救助人口扩大到９万人，完成扶贫搬迁１万人，改造农村危房２０００户。（４）将失地困难农民纳入城乡社会救助保障范围。（５）解决农村群众安全饮水５万人，启动解决市城郊居民饮用“毛水”工程，新建通村油（水泥）路２０００公里。（６）城区启动新建一所初中、扩建柳林小学，缓解中心区学生入学难问题。（７）农村新型合作医疗参合率平均达到８５％以上，城区实现社区卫生服务全覆盖，办好惠民医院试点。（８）进一步加快污水治理步伐，启动泗河、竹山、竹溪、武当山等污水处理厂建设。（９）实施堰中蔬菜批发市场二期工程建设，建设农村超市和农资放心店各１２０个。（１０）建设十堰５００千伏输变电网工程，加快推进天然气利用项目建设。

各位代表！新的一年，政府工作任务繁重，我们深感责任重大。全市人民对政府的信任和支持，给了我们克难奋进的力量和信心。我们坚信，在市委的正确领导下，在市人大、政协的监督支持下，有全市人民的共同努力，十堰一定能够实现经济社会又好又快的发展！
